# Oral and non-oral lichen planus show genetic heterogeneity and differential risk for autoimmune disease and oral cancer

**DOI:** 10.1016/j.ajhg.2024.04.020

**Published:** 2024-05-21

**Authors:** Mary Pat Reeve, Mari Vehviläinen, Shuang Luo, Jarmo Ritari, Juha Karjalainen, Javier Gracia-Tabuenca, Juha Mehtonen, Shanmukha Sampath Padmanabhuni, Nikita Kolosov, Mykyta Artomov, Harri Siirtola, Hanna M. Olilla, FinnGen, Daniel Graham, Jukka Partanen, Ramnik J. Xavier, Mark J. Daly, Samuli Ripatti, Tuula Salo, Maria Siponen

**Affiliations:** 1 Institute for Molecular Medicine Finland (FIMM), Helsinki Institute of Life Science, University of Helsinki, Helsinki, Finland; 2 Broad Institute of MIT and Harvard, Cambridge, MA, USA; 3 Department of Oral and Maxillofacial Diseases, Faculty of Medicine, University of Helsinki, Helsinki, Finland; 4 Finnish Red Cross Blood Service, Helsinki, Finland; 5 Institute for Genomic Medicine, Nationwide Children’s Hospital, Columbus, OH, USA; 6 Ohio State University College of Medicine, Columbus, OH, USA; 7 TAUCHI Research Center, Tampere University, Tampere, Finland; 8 Center for Genomic Medicine, Massachusetts General Hospital, Harvard Medical School, Boston, MA, USA; 9 Anesthesia, Critical Care, and Pain Medicine, Massachusetts General Hospital and Harvard Medical School, Boston, MA, USA; 10 Center for Computational and Integrative Biology, Massachusetts General Hospital and Harvard Medical School, Boston, MA 02114, USA; 11 Department of Molecular Biology, Massachusetts General Hospital and Harvard Medical School, Boston, MA 02114, USA; 12 Klarman Cell Observatory, Broad Institute of MIT and Harvard, Cambridge, MA 02142, USA; 13 Analytical and Translational Genetics Unit, Massachusetts General Hospital and Harvard Medical School, Boston, MA 02114, USA; 14 Research Unit of Population Health, Department of Oral Pathology, University of Oulu and Oulu University Hospital, Oulu, Finland; 15 Medical Research Center, Oulu University Hospital, Oulu, Finland; 16 Department of Oral and Maxillofacial Diseases, and Translational Immunology Program (TRIMM), University of Helsinki, Helsinki, Finland; 17 Institute of Dentistry, Faculty of Health Sciences, University of Eastern Finland, Kuopio, Finland; 18 Odontology Education Unit, and Oral and Maxillofacial Diseases Clinic, Kuopio University Hospital, Kuopio, Finland

**Keywords:** lichen planus, oral lichen planus, hypothyroidism, autoimmune disease, genetics, oral cancer

## Abstract

Lichen planus (LP) is a T-cell-mediated inflammatory disease affecting squamous epithelia in many parts of the body, most often the skin and oral mucosa. Cutaneous LP is usually transient and oral LP (OLP) is most often chronic, so we performed a large-scale genetic and epidemiological study of LP to address whether the oral and non-oral subgroups have shared or distinct underlying pathologies and their overlap with autoimmune disease. Using lifelong records covering diagnoses, procedures, and clinic identity from 473,580 individuals in the FinnGen study, genome-wide association analyses were conducted on carefully constructed subcategories of OLP (*n* = 3,323) and non-oral LP (*n* = 4,356) and on the combined group. We identified 15 genome-wide significant associations in FinnGen and an additional 12 when meta-analyzed with UKBB (27 independent associations at 25 distinct genomic locations), most of which are shared between oral and non-oral LP. Many associations coincide with known autoimmune disease loci, consistent with the epidemiologic enrichment of LP with hypothyroidism and other autoimmune diseases. Notably, a third of the FinnGen associations demonstrate significant differences between OLP and non-OLP. We also observed a 13.6-fold risk for tongue cancer and an elevated risk for other oral cancers in OLP, in agreement with earlier reports that connect LP with higher cancer incidence. In addition to a large-scale dissection of LP genetics and comorbidities, our study demonstrates the use of comprehensive, multidimensional health registry data to address outstanding clinical questions and reveal underlying biological mechanisms in common but understudied diseases.

## Graphical abstract

**Figure:**
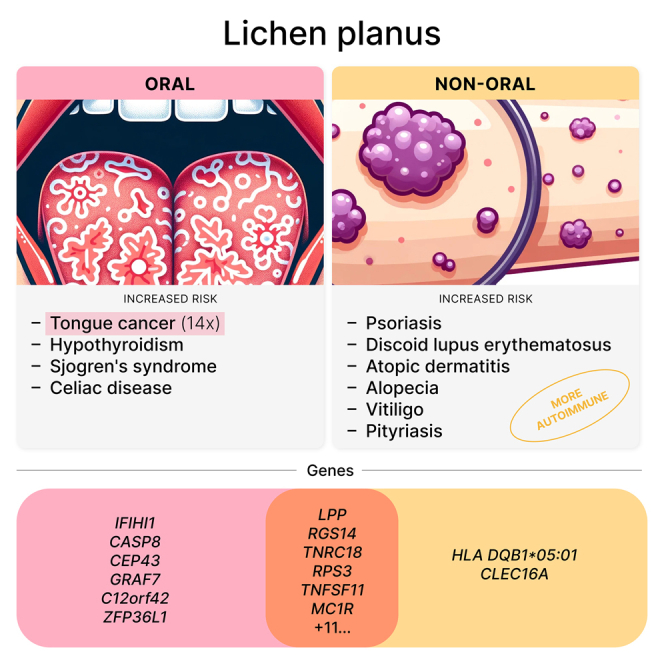

## Teaser

The relationship of oral and skin forms of lichen planus (LP) is not understood. Using GWASs in two biobanks, we identify 27 independent hits, which include shared and distinct genetic features across clinical presentations and highlight an immune-mediated etiology to LP. Notably, oral LP shows 14-fold risk for tongue cancer.

## Introduction

Lichen planus (LP) is a chronic inflammatory and immune-mediated disease affecting stratified squamous epithelia. LP is considered a T-cell-mediated disease, in which CD4^+^ and CD8^+^ T cells accumulate in the dermis, while CD8^+^ T cells infiltrate the epidermis leading to an interface dermatitis.^1^ LP can affect the skin (cutaneous LP), the scalp (lichen planopilaris), nails (lichen unguis), and/or mucous membranes (penile or vulvar LP, oral LP, or esophageal LP).^1^^,^^2^ Many forms of LP can cause pain and discomfort and, unfortunately, only symptomatic treatment exists.

Of the most common LP forms, cutaneous LP is estimated to occur in 0.2%–1%^3^^,^^4^ while OLP affects about 0.1%–3.2% of the adult population.^5^^,^^6^ All forms of LP can occur at any age but are most common in middle-aged adults.^1^ No known sex bias or ancestry predisposition is evident in the cutaneous form, whereas with OLP, 60%–75% of individuals are female.^1^ LP is associated with an increased risk of cancer of the lip, tongue, oral cavity, esophagus, larynx, and vulva,^7^ with a malignant transformation risk of OLP of roughly 1%–1.5%.^8^^,^^9^^,^^10^ Notably, LP skin lesions tend to be transient and resolve; in contrast, OLP is usually chronic and spontaneous complete remission is rare.^11^

The precise etiology of LP is not well understood, but a genetic contribution has been suggested by numerous reports implicating HLA alleles associated with LP, for example, *HLA-A3* (cutaneous LP, reticular OLP), *HLA-A5*, *HLA-A9* and *B8* (erosive OLP), *HLA-Bw57* (OLP in English individuals), *HLA-DR1*, *HLA-DR6* (HCV-associated oral LP), *HLA-DR9* (OLP in Japanese and Chinese individuals), *HLA-DRB1^∗^0101* (cutaneous/oral LP), *DQB1^∗^0201* (vulvovaginal gingival syndrome), *HLA DRB1∗11*, and *DQB1∗03* alleles (lichen planopilaris).^12^^,^^13^^,^^14^^,^^15^^,^^16^^,^^17^^,^^18^^,^^19^^,^^20^^,^^21^^,^^22^^,^^23^ Outside the MHC, several candidate gene studies and several systematic meta-analyses of such studies^24^^,^^25^ have been performed in recent years suggesting potential associations at *IFNG, IL18*, and *IL10*. These studies, however, and broader genome-wide studies have been extremely limited in sample size and unable to provide conclusively genome-wide significant findings outside the MHC.

Epidemiologically more has been described and LP has been reported in association with many other diseases, potentially providing further clues. Such observations include overlap with thyroid diseases,^26^ dyslipidemia,^26^ hepatitis C virus infection,^27^^,^^28^^,^^29^^,^^30^ and hepatitis B vaccination.^31^ Other conditions with altered immunity, especially vitiligo, alopecia areata, ulcerative colitis, morphea, and myasthenia gravis, have been reported as more prevalent among LP-affected individuals.^1^ Studies looking at the association of diabetes have been contradictory but in a recent meta-analysis a significant association was seen between OLP and diabetes mellitus.^32^^,^^33^ In two meta-analyses^24^^,^^33^ OLP has shown a significant association with thyroid disease and hypothyroidism specifically.

Given the lack of genetic studies of LP to date, we sought to take advantage of the FinnGen project, which integrates health history—including diagnostics from a range of relevant dental and dermatological clinics—with genome information from more than 473,000 individuals. With this resource, we performed a well-powered genome-wide association study of oral and non-oral LP as well as a meta-analysis of both and identified a total of 27 genome-wide significant associations with LP, ten of which are also conclusively associated with hypothyroidism and other immune-mediated diseases and nine of which overlap with loci mapped for eosinophil levels.

## Material and methods

### Study cohort

The FinnGen study (https://www.finngen.fi/en) is a public-private partnership including Finnish universities, biobanks, and hospital districts together with several pharmaceutical companies; it was founded in the year 2017. The aim is to collect both National Health Record and genetic data from 500,000 Finns. The study participants include individuals with acute and chronic diseases, healthy volunteers, and population collections. R11 consists of 473,580 individuals (55% females and 45% males).

### FinnGen Ethics Statement

Individuals in FinnGen provided informed consent for biobank research, based on the Finnish Biobank Act. Alternatively, separate research cohorts, collected prior the Finnish Biobank Act came into effect (in September 2013) and start of FinnGen (August 2017), were collected based on study-specific consents and later transferred to the Finnish biobanks after approval by Fimea (Finnish Medicines Agency) and the National Supervisory Authority for Welfare and Health. Recruitment protocols followed the biobank protocols approved by Fimea. The Coordinating Ethics Committee of the Hospital District of Helsinki and Uusimaa (HUS) statement number for the FinnGen study is Nr HUS/990/2017. Further details on permit and biobank decision numbers are available in the FinnGen Ethics Statement in the supplemental information.

### Phenotype definitions

**Figure 1:**
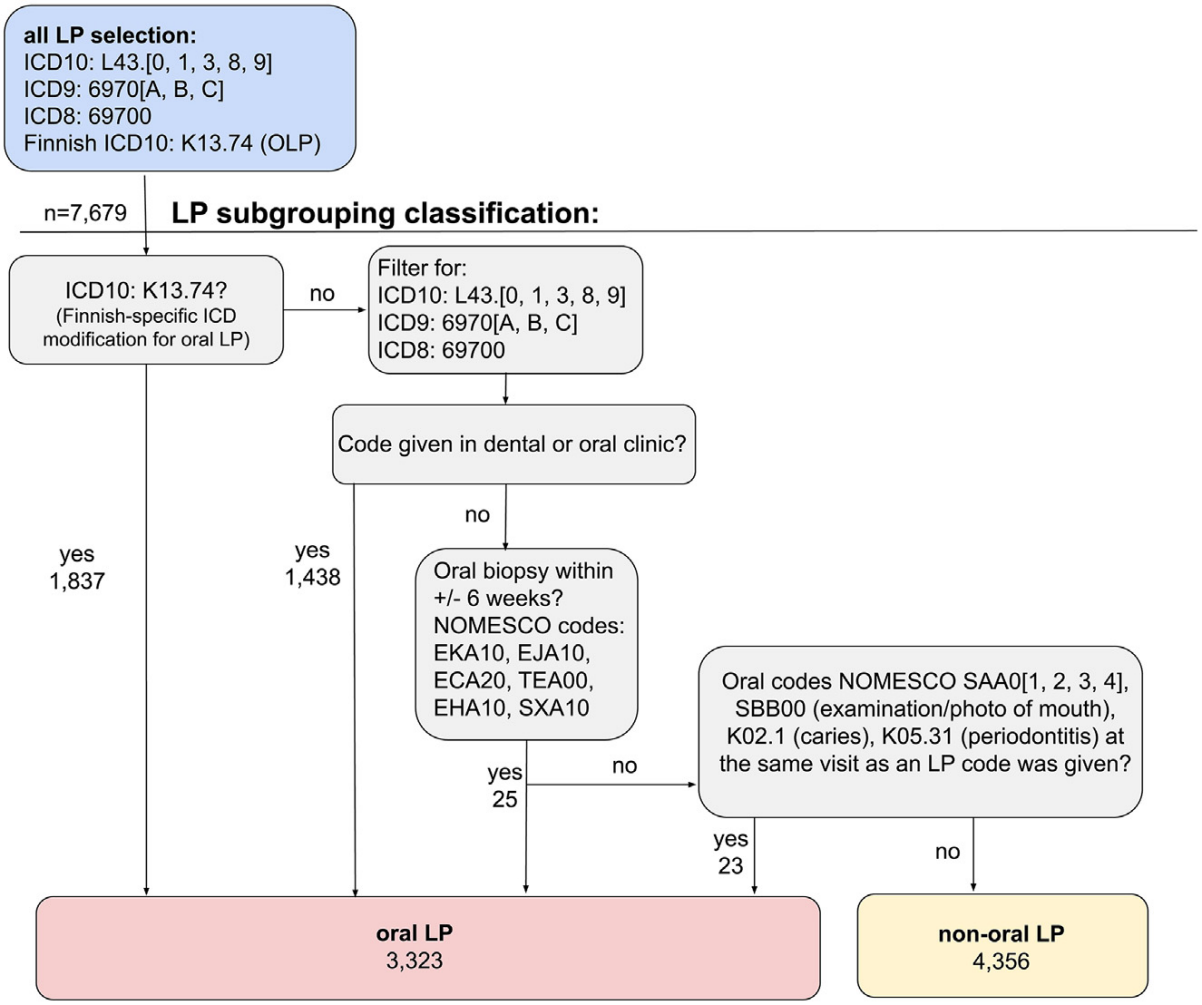
Categorization of lichen planus subgroups in FinnGen

ICD codes for lichen planus-L43 (ICD10 with extensions 0,1,3,8,9) and 6970 (ICD 8 and 9 with Finnish extensions A, B, C) do not indicate physical location, but we inferred those individuals diagnosed in oral-related clinics would mainly occur if it were seen in the mouth. An additional 25 individuals with LP ICD codes were labeled oral based on registry data for oral biopsies (often used to confirm presumed LP diagnosis) and 23 individuals based on oral-related codes at the same visit (Figure 1).

Validation of this strategy can be examined by looking at unselected codes. For example, Finnish ICD10 code K13.78 for “Other changes in the oral mucosa” is seen in 357/3,323 oral vs. 79/4,356 in non-oral LP individuals (oral vs. non-oral OR = 6.5, *p* = 7.0 × 10^−65^). By contrast, non-oral individuals show significantly increased rates of topical treatment (mometasone, OR = 2.0, *p* = 3.8 × 10^−49^) and biopsies of skin and subcutaneous tissue (OR = 2.3, *p* = 3.1 × 10^−34^). Other oral diagnostic codes for differential diagnoses of OLP (e.g., diagnosis of leukoplakia or oral candidiasis) were in strong excess in oral versus non-oral LP; however, as there were still a handful of these diagnoses in the non-oral group, we might expect that there could be some oral individuals among those specified as non-oral.

Inclusion of FinnGen study samples in the all-LP group was based on ICD codes (ICD-10: L43, L43.[0,1,3,8,9]; Finnish-specific oral ICD-10 K13.74; ICD-9 codes 6970[A,B,C]; ICD-8 69700). After initial selection, individuals were put in the oral subgroup if the LP diagnosis was given by an oral-related clinic (dental, orthodontic, oral surgery), if they had an oral biopsy as defined by the Nordic Medico-Statistical Committee (NOMESCO) procedure codes EKA10, EJA10, EHA10, ECA20, TEA00, or SXA10 within ±6 weeks of LP code, or if they had an oral examination, photography of the mouth and dentition, caries, or periodontitis code at the same visit as the LP diagnosis (Figure 1). All other individuals were considered non-oral. Logically, as the oral LP group is strictly defined and non-oral assignment is by exclusion, there may be a handful of individuals diagnosed with oral LP by a general practitioner who did not use the oral LP code or who have cutaneous LP but later develop OLP who are in the current non-oral grouping.

**Figure 2:**
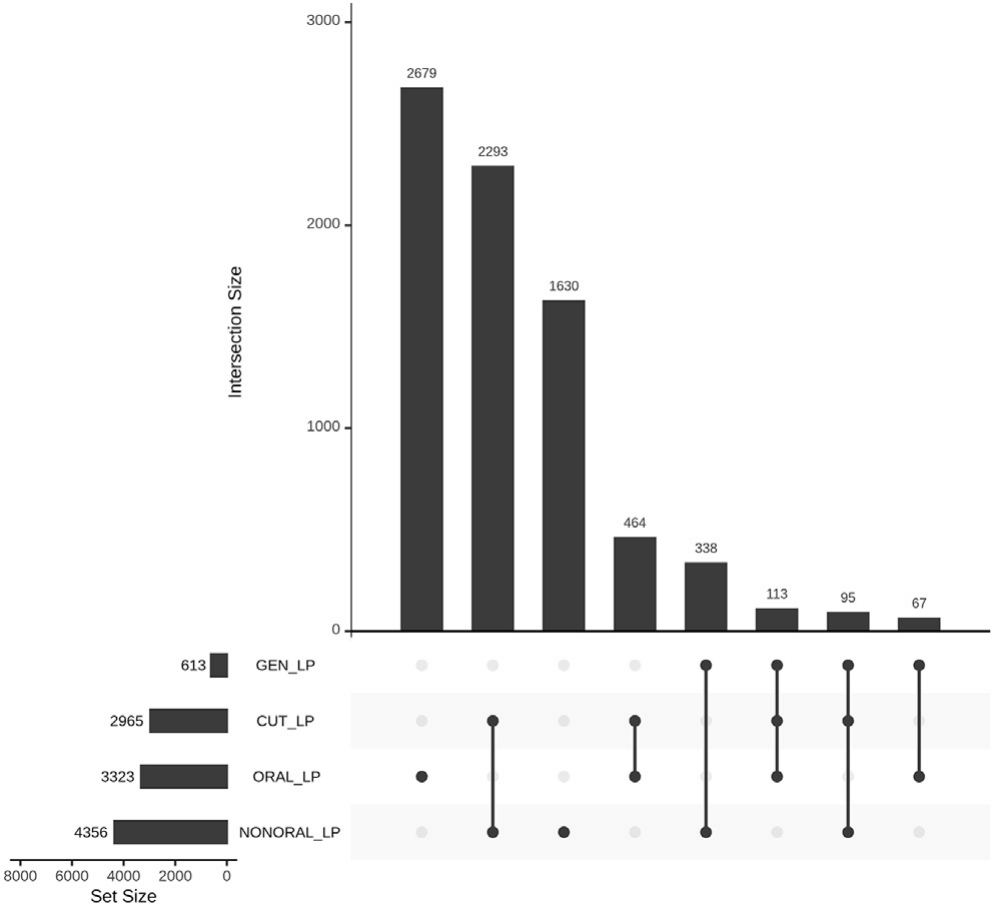
UpSet plot showing the intersection of genital LP (GEN_LP) and cutaneous LP (CUT_LP) with the oral (ORAL_LP) and non-oral (NONORAL_LP) subgroups Intersections with fewer than five individuals are not displayed due to data-protection regulations.

Since the principal ICD codes for LP (e.g., L43) are not body location specific, we cannot create exact subgroups; additionally, individuals may have more than one type of LP during their lifetime. To understand the magnitude of possible overlapping LP subgroupings, we additionally grouped individuals as cutaneous LP if seen at a dermatologist and genital LP if a gynecologist or urologist saw them. If individuals had the Finnish site-specific code for vaginal LP (N77.8^∗^L43.9) and no other LP codes, they were not included in any analyses but are provided as an UpSet plot (Figure 2). Of the individuals classified as OLP, 577/3,323 (17.4%) were also seen at a dermatological clinic. Of the non-OLP individuals, 10.2% (443/4,356) have been seen for genital LP, and of the OLP group, 5.4% (180/3,323).

**Figure 3:**
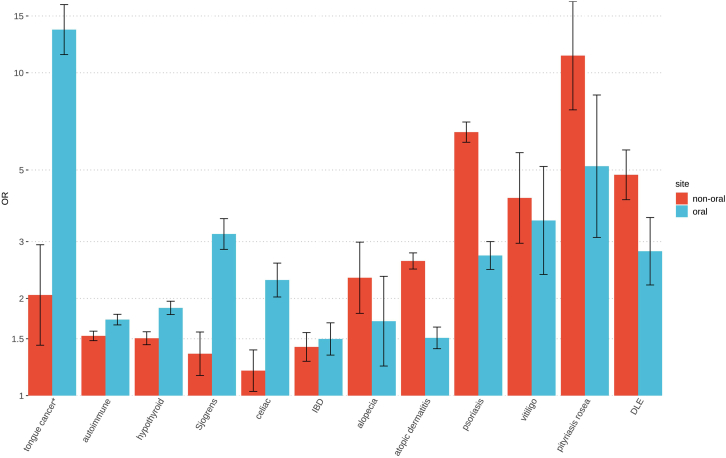
Excess comorbidities between LP oral and non-oral subgroupings and other diseases in FinnGen Error bars represent 95% CI on the estimated effect. (^∗^243 OLP-affected individuals with a leukoplakia diagnosis within one month have been removed from the tongue cancer analysis.) Data are presented in Table S1.

As the differential diagnosis of OLP can be challenging and may be initially misdiagnosed as oral leukoplakia (which also carries a documented cancer risk^34^), we also examined the occurrence of leukoplakia in OLP. While registry data are limited in terms of access to full results of histopathology, OLP was diagnosed in specialty dental clinics in 93.8% (3,117/3,323) of OLP-affected individuals, and biopsies were performed as part of the diagnostic procedure in 45.4% (1,510/3,323) of OLP-affected individuals. Standard diagnostic procedure in Finland is such that oral symmetric reticular lesions do not require biopsy for OLP diagnosis but if lesions are atypical or the diagnosis is otherwise uncertain, biopsies will be taken for confirmation. With the majority of individuals being diagnosed by experts, this is a highly confident definition to take forward into the genetic study. Nonetheless, it is possible that during the diagnostic process, initial codings may later be replaced with a different diagnosis after further testing such that registry data are certainly imperfect. For example, we identified 243 individuals in the OLP subgroup who also received a diagnosis of leukoplakia within a month of their LP diagnosis. Since both of these diagnoses have high rates of cancer transformation, these individuals with concomitant OLP and leukoplakia diagnoses were not included in oral cancer risk calculations (Figure 3) so as to make this strictly about progression from OLP.

The full set of 4,962 FinnGen DF11 endpoints were analyzed for phenotypic risk assessments. All FinnGen DF11 endpoint definitions can be found from https://www.finngen.fi/en/researchers/clinical-endpoints.

Endpoints shown in Figure 3 are specifically: C3_TONGENAS, AUTOIMMUNE, E4_HYTHY_AI_STRICT, M13_SJOGREN, K11_CELIAC, K11_KELAIBD, L12_ALOPECAREATA, L12_ATOPIC, L12_PSORIASIS, L12_VITILIGO, L12_PITYROCHEA, L12_LUPUS; the medical code definitions can be found in https://risteys.finngen.fi. Logistic regression (R 4.1.3, glm family = binary) adjusted for age, age^2^, and sex was used to calculate OR between OLP and non-OLP subgroupings and all FinnGen DF11 core endpoints.

To define UKBB individuals with LP, we used individuals with ICD10 codes beginning L43 and included individuals from both inpatient and outpatient clinics. In UKBB we could not classify subgroups based on clinic type.

### HLA imputation

HLA alleles were imputed using HIBAG v.1.18.1^35^ R library with a Finnish population-specific HLA reference panel (*n* = 1,150) based on ∼ 4,500 variants within the MHC region (chr6:28.51–33.48 Mb; hg38/GRCh38) where posterior imputation probabilities were considered when calculating imputed allele dosages. Association of imputed HLA alleles with OLP and non-OLP was calculated with PLINK^36^ (v.1.90b6.26) assuming additive effects of allele dosage and run in stepwise fashion adjusting in the second run for the dominant *DQB1^∗^05:01* haplotype.

### Genotype data QC and association testing

Array-based genotype data in FinnGen were called and subjected to variant and sample level quality control (QC) followed by phasing and imputation as described in Kurki et al.^37^ In FinnGen DF11, this project-wide process resulted in a total of 453,733 individuals after removal of related individuals and non-Finnish ancestry persons and were used in all FinnGen analyses. FinnGen data analysis pipelines are freely available in https://github.com/FINNGEN/; the FinnGen Handbook, https://finngen.gitbook.io/documentation/, contains a detailed description of data production and analysis, including code used to run analyses.

Of the 7,679 LP individuals, 7,428 passed all genotype QC and inclusion criteria and were compared with 404,662 control subjects. GWAS analysis was performed using REGENIE 2.2.4^38^ using a logistic mixed model adjusted for age, sex, genotyping batch, and the first ten principal components of ancestry with an approximate Firth test for robust effect size estimation. All variants reaching a genome-wide significance *p* value threshold of 5 × 10^−8^ are considered as genome-wide significant (GWS), and all disease endpoints reaching multiple testing corrected (for the 4,975 endpoints tested) *p* value threshold of 0.05/4,975 = 1.0 × 10^−5^ were considered as phenome-wide significant (PWS). To confirm independence of associations in two regions, fine-mapping of loci within FinnGen was performed using SuSIE^39^ as well as directly by rerunning REGENIE with the index variant incorporated as a covariate. Such analyses were run on a 3-Mb interval centered on the index variant as previously described.^37^ Both variants at *LPP* were GWS in FinnGen and remained so upon full conditional analysis; *IFIH1* associations were not attenuated upon conditional analysis and both have been previously demonstrated to be independently associated to autoimmune disease.

To extend and replicate FinnGen findings, inverse variance weighted meta-analysis with the UK Biobank was performed using a custom pipeline available at https://github.com/FINNGEN/META_ANALYSIS/. UKBB summary statistics are available at http://pan.ukbb.broadinstitute.org and GRCh37 to 38 liftover was performed using Picard software (https://gatk.broadinstitute.org/hc/en-us/articles/360037060932-LiftoverVcf-Picard-)—all steps performed as previous integration between the two.^37^ The Open Targets Genetics site (February 2022, v.22.02)^40^ was used to assess prior evidence of association to phenotype, expression, and proteomic publication.

### Additional statistical tools used for phenotypic and genotypic analyses

Differences in baseline demographics and clinical characteristics were tested using χ^2^ tests in R 4.1.3.

To calculate medical code overrepresentation near to the initial lichen planus diagnosis, we selected 50 birth year- and sex-matched control subjects for each individual with an LP phenotype and measured OHDSI categorical excess at the initial visit, ±2 weeks of the initial visit, and ±1-year increments going five years in either direction. To test for significance and calculate OR, Fisher’s exact test from R 4.1.3 was used.

Genetic correlations were calculated from summary statistics using ldsc.^41^

### PGS browser

We derived 129 polygenic score (PGS) models from external genome-wide association studies (GWASs) using PRS-CS-auto (https://github.com/FINNGEN/CS-PRS-pipeline). As an LD reference panel, we utilized 503 European individuals from the 1000 Genomes project and HapMap3 variants.^42^ Next, each PGS model was used to calculate individual PGS scores for 430,897 FinnGen Data Freeze 10 individuals using PLINK 2.0 –score function.^36^^,^^43^ To investigate the relationship between subgroupings and the 129 PGSs, we conducted logistic regression analyses for each PGS individually. The models were adjusted for age, sex, principal components 1–10, genotyping batch, and baseline year. Individuals lacking any of these covariates were excluded from the analysis, resulting in a total of 392,650 individuals.

All analyses were conducted using the GUI of the PGS browser, which is currently available as an internal tool for all FinnGen Trusted Research Environment (TRE) users.

## Results

### Defining oral vs. non-oral lichen planus based on electronic health data

Based on the International Classification of Diseases (ICD) diagnosis codes (versions 8, 9, and 10) assigned by clinicians in Finland, we identified a total of 7,679 LP-affected individuals in FinnGen Data Freeze 11 (DF11) (cohort prevalence of 1.6%). 465,900 individuals with no ICD-based diagnosis of LP were used as control subjects. In UKBB we identified an additional 1,998 individuals with LP (cohort prevalence 0.48%) and 418,544 control subjects. Both datasets are within previously reported ranges of 0.1%–3.2% prevalence^5^^,^^6^; higher prevalence in FinnGen is likely due to extensive information from dental clinics in Finland that is not available in UKBB.

We used the deep electronic health registry data in FinnGen DF11 to create oral (*n* = 3,323) and non-oral (*n* = 4,356) LP subgroupings. We validated the distinction between oral and non-oral by comparing detailed medical coding data not used to create the classification. Comparing the oral and non-oral LP groups, we observed statistically significant excesses of oral indications not selected for in the OLP subgroup, while in non-OLP topical medications and cutaneous skin biopsies were seen significantly more often (see material and methods). In UKBB we could not create a classification of oral and non-oral LP subgroups from the clinic data available to us.

The FinnGen LP cohort is significantly enriched for females (67.4% of LP-affected individuals are female versus 56.1% of FinnGen participants overall) with the oral subgroup showing an even greater excess over non-oral (74.0% OLP- vs. 62.4% non-OLP-affected individuals are female, *p* = 1.66 × 10^−26^). The median age of onset in FinnGen for oral and non-oral groups is 60.6 years. In UKBB we find good concordance with 68.7% of UKBB LP-affected individuals female versus 54.1% of UKBB participants overall, and the median age of onset in UKBB is 61.0.

### Phenotypic correlations in FinnGen

To explore disease overlaps with LP in FinnGen, the oral, non-oral, and total LP groups were also compared at the level of FinnGen endpoints. These endpoints combine ICD 8, 9, and 10 codes with hospital procedural codes to create more comprehensive phenotypes covering a broad range of more than 4,000 diseases (all definitions available at https://risteys.finregistry.fi/). To remove any shared cancer risk with leukoplakia,^34^ we removed the 243 OLP-affected individuals with a leukoplakia diagnosis ±1 month from OLP in the phenotypic correlations. The OLP group minus leukoplakia carries an OR of 13.6 (CI 9.6–19.3) (*n* = 403, *p* = 1.6 × 10^−48^) for risk of tongue cancer and is the most notable of the phenotypic correlations seen specifically in OLP. Significantly increased risk for oral cancers in other, rarer sites, including cancer of the gingiva (*n* = 111, OR 16.6 [CI 9.2–29.8], *p* = 9.5 × 10^−21^) and lip (*n* = 231, OR = 5.0 [CI 2.3–10.6], *p* = 3.4 × 10^−5^), were also seen for the adjusted OLP subgroup; however, the small number of individuals precludes accurate risk estimates for these. Using the longitudinal data in FinnGen, we find OLP has a 10-year oral cancer transformation rate (first diagnosis of oral cancer 0–10 years after initial OLP diagnosis) of 1.9% (63/3,323) compared to a base rate of 0.2% for oral cancer across the entire FinnGen cohort. As shown in Figure 3, both oral and non-oral forms of LP show broadly increased risk of autoimmune and inflammatory disease. The oral group has a somewhat greater risk for autoimmune hypothyroidism, celiac disease, and Sjögren’s syndrome, and the non-oral group shows a higher risk for discoid lupus erythematosus (DLE), psoriasis, and atopic dermatitis. Both oral and non-oral LP demonstrated similarly increased risk for inflammatory bowel disease (IBD), alopecia areata, and vitiligo.

We used a time-based analysis to examine the events' order near the first lichen planus diagnosis across all individuals. To increase the resolution of events, we used the Observational Health Data Sciences and Informatics (OHDSI) common database model (CDM) where each medical visit’s data in FinnGen is searchable as drug exposures, condition occurrences, procedure occurrences, measurements, and observations. Fifty birth year- and sex-matched control subjects were selected for each case and used to look at which OHDSI categories are overrepresented at the initial visit, ±2 weeks from the initial visit, and ±1-year increments five years before and after.

At the same visit with the LP diagnosis, many other differential diagnoses are seen at significantly higher rates in LP-affected individuals than matched control subjects (*p* < 1 × 10^−5^ and OR > 2) including tinea pedis (ringworm), pruritic rash, candidiasis, atopic dermatitis, rosacea, erysipelas, stomatitis, actinic keratosis, psoriasis, bullous pemphigoid, seborrheic keratosis, and infective dermatitis. Also at the initial diagnosis, we see significant excess of biopsies of the lip, palate, gingiva, cheek, mouth, floor of the mouth, tongue, skin, subcutaneous tissue, vulva, perineum, vagina, endometrium, and cervix. Topical ointments, corticosteroids, and antifungals are also significant in the original time point (Table S2).

Lichenoid reactions to medications^44^ and medical procedures^45^ have also been reported. However, in the two weeks preceding the initial diagnosis, we do not observe significant excesses of medications or procedures besides the expected enrichment of consultations, imaging, and topical medications (such as desonide, clobetasol, and betamethasone). Expanding to one year preceding the initial visit for all LP-affected individuals, we see higher rates of acyclovir prescription (*n* = 95, OR = 2.2, *p* = 1.46 × 10^−11^) indicating a possible viral infection, and increased scabies infection (*n* = 24, OR = 6.5, *p* = 6.3 × 10^−12^). In the year preceding diagnosis of OLP, there is a significant excess of candidiasis diagnoses (*n* = 31, OR = 17, *p* = 1.1 × 10^−25^) and excess prescription of numerous antifungals such as fluconazole, amphotericin B, miconazole, and nystatin.

### Genomic loci associated with lichen planus and subgroupings of LP

Using the data freeze analysis pipelines of the FinnGen project^37^ (see material and methods), we performed case-control GWASs of all individuals with LP in FinnGen DF11 and observed 12 independent associations associated with lichen planus at genome-wide significance (*p* < 5 × 10^−8^). Additionally, we analyzed LP in the UKBB using both inpatient and outpatient data. The UKBB scan showed two genome-wide significant hits—the very strong association to the HLA region and the *CEP43* locus also seen in FinnGen. Meta-analysis of FinnGen and UKBB revealed an additional 13 loci at genome-wide significance (Table 1, expanded data in Table S3). The two studies did not have significant heterogeneity at any of these associations. The loci containing *IFIH1* and *LPP* contain two independent associations as assessed by both summary statistic fine-mapping and genotype-based conditional analysis in FinnGen (see material and methods). Of note, we find no support for suggestive associations from candidate gene meta-analyses^24^^,^^25^ which highlighted specific variants at *IFNG* (rs2430561), *IL18* (rs187238), and *IL10* (rs1800872) (all betas <0.01, *p* > 0.05).

**Table 1:** Genetic associations in the FinnGen and FinnGen-UKBB meta-analysis and each LP subgrouping

									**Blood cell counts****^40^**				**Disease overlaps**							
**Most severe**	**Min *p*_val_**	**Beta**	**Variant**	**Nearest gene**	**Heterogeneity**	***cis* eQTL**	***cis* pQTL**	***cis* sQTL**	**Eosinophils**	**Lymphocytes**	**Neutrophils**	**White blood cells**	**IBD**^46^	**Hypothyroidism****^37^**	**Vitiligo****^37^**	**Basal cell carcinoma**^47^	**Atopic dermatitis****^37^**	**Celiac****^37^**	**Psoriasis****^37^**	**T1D**^48^
Intergenic	4.2E−10	0.09	rs2111485	*IFIH1*	OLP	–	–	–	⏊	⏊	⏊	⏊	⏊	∥	⏊	–	–	–	∥	∥
**Missense**	1.9E−10	−0.37	rs35667974	*IFIH1*	–	–	–	–	–	⏊	–	⏊	⏊	∥	–	–	–	–	∥	∥
Intronic	4.6E−08	0.38	rs7571586	*CASP8*	OLP	–	–	–	–	–	–	–	–	–	–	–	–	–	–	–
Intronic	1.5E−16	−0.12	rs6780858	*LPP*	–	–	–	–	–	⏊	–	–	–	∥	∥	⏊	–	∥	–	–
Intronic	4.3E−10	−0.12	rs56328339	*LPP*	–	–	–	–	∥	–	–	–	∥	–	–	–	–	–	–	–
Intronic	9.4E−09	0.10	rs67111717	*RGS14*	–	↓↑*RGS14*	↑*LMAN2*	↓*RGS14*	∥	–	∥	–	∥	–	–	–	∥	–	∥	–
HLA	7.5E−211	0.53	rs28592859	*HLA-DQB1^∗^05:01*	non-OLP	–	–	–	–	–	–	–	–	–	–	–	–	–	–	–
3′ UTR	3.3E−21	0.14	rs9459853	*CEP43*	OLP	↑*CEP43*	↓*RNASET2*	–	–	–	–	–	∥	∥	∥	⏊	–	–	–	–
Intronic	3.5E−10	0.26	rs748670681^∗^	*TNRC18*	–	–	–	–	–	–	–	–	∥	⏊	–	–	–	–	∥	–
Intergenic	2.4E−08	−0.17	rs11988402	*GFRA2*	OLP	–	–	–	–	–	–	–	–	–	–	–	–	–	–	–
Intronic	1.0E−08	1.39	rs1192863020^∗^	*RPS3*	–	–	–	–	–	–	–	–	–	–	–	–	–	–	–	–
Intergenic	5.8E−13	−0.21	rs10861030	*C12orf42*	OLP	–	–	–	–	–	–	–	–	∥	–	–	–	–	–	–
Intronic	5.7E−18	0.13	rs2093816	*TNFSF11*	–	↓*TNFSF11*	–	–	∥	⏊	–	–	–	∥	–	–	–	–	–	–
Intronic	6.2E−11	−0.16	rs193769	*CLEC16A*	non-OLP	–	–	–	–	–	–	–	–	–	–	–	–	–	–	–
Intronic	2.0E−11	0.10	rs4328441	*MC1R*	–	–	↑*DPEP1*	–	–	–	–	–	–	–	∥	⏊	–	–	–	–

**Additional GWS hits from FG-UKBB meta**																				

Intergenic	8.0E−11	−0.12	rs35318359	*IL18RAP*	–	↑↓*IL18RAP*	↓*IL18R1*, ↓*IL1RL1*, ↓*IL1RL2*	*↑IL18RAP*	–	–	–	–	–	–	–	–	∥	∥	–	–
Intronic	1.8E−08	−0.09	rs1432611	*FOXP1*	–	–	–	–	–	–	–	–	–	–	∥	⏊	–	–	–	–
Intronic	8.0E−09	0.09	rs3755570	*ST3GAL6*	–	↑*ST3GAL6*	↑*ST3GAL6*	–	∥	–	–	–	–	∥	–	⏊	–	–	–	–
Intronic	2.6E−09	0.09	rs1391440	*TET2*	–	↑*TET2*	–	–	–	⏊	∥	∥	–	–	–	–	–	–	–	–
Intronic	7.9E−09	0.09	rs2927608	*ERAP2*	–	↑*ERAP2*, ↑*LNPEP*	↑*ERAP2*, ↓*ERAP1*	↑↓*ERAP2*, ↑↓*LNPEP*	–	–	∥	∥	∥	–	–	–	–	–	–	–
**Missense**	3.4E−09	0.22	rs117744081	*CPVL*	–		↓*CPVL*	–	–	–	–	–	–	–	∥	⏊	–	–	–	–
Intergenic	2.6E−08	0.08	rs7096384	*IL2RA*	–	↑*IL2RA*	↑*RBM17*	–	∥	∥	–	–	∥	∥	–	–	–	–	–	∥
Intronic	7.8E−10	0.10	rs2893907	*ZNF365*	–	–	–	–	∥	–	–	–	⏊	–	–	–	∥	–	–	–
Intronic	1.3E−08	0.10	rs10745680	*PLXNC1*	–	–	↑*PLXNC1*	–	∥	–	–	–	–	–	–	–	–	–	–	–
5′ UTR	4.3E−09	−0.09	rs3825568	*ZFP36L1*	OLP	↑*ZFP36L1*	↑↓*ZFP36L1*	–	–	–	–	–	∥	–	–	–	–	–	–	–
Intronic	2.2E−08	−0.08	rs2601191	*IQGAP1*	–	↑*IQGAP1*	–	↑*IQGAP1*	–	⏊	–	–	–	–	–	–	–	–	–	–
**Missense**	1.9E−08	−0.23	rs112301322	*IKZF3*	–	↑*IKZF3*, ↑↓*GSDMB*	–	↑↓*GSDMB*	∥	–	∥	⏊	–	∥	–	–	–	–	–	–

Finnish-enriched (FE) variants seen at >100× frequency in FinnGen and therefore not in the UKBB are indicated with an asterisk (^∗^). Loci demonstrating significant heterogeneity between the oral and non-oral are indicated in the “heterogeneity” column. Colocalized eQTLs, pQTLs, and sQTLs and direction of regulation are also shown. *cis*-eQTLs with double arrows represent instances where changes of isoform balance, rather than unidirectional up- or down-regulation, have been reported in eQTLCatalogue. Loci included as overlapping with other diseases include only those achieving genome-wide significance in the second phenotype; **∥** indicates the same direction of effect, and ⏊ indicates opposite directions of effect. HLA is not included in the overlap counts because of its pervasive association to nearly all traits and the lack of systematic fine-mapping in published studies.

**Figure 4:**
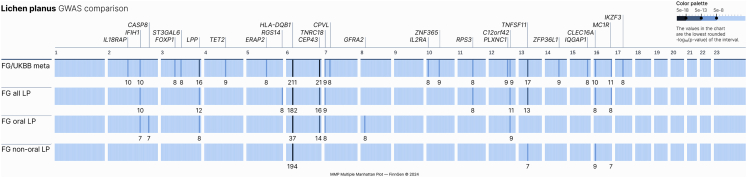
Genome-wide significant loci from meta-analysis of UKBB-FG, FG all LP, FG OLP, and FG non-OLP Traditional Manhattan plots aligned are available as Figure S1.

We additionally performed GWASs on the OLP and non-OLP subgroupings within FinnGen to explore the genetic similarity between them. OLP and non-OLP both produced multiple genome-wide significant hits in addition to the MHC (Figure 4). Most are coincident with hits found in the all-LP analyses, though two are uniquely genome-wide significant in the OLP subset and are included in Table 1 for completeness, bringing the total number to 27 independent associations at 25 genomic loci (expanded data in Table S3). A test of heterogeneity of effect from an inverse-variance weighted meta-analysis of the two subgroupings indicated that eight of the 27 total associations demonstrated significant heterogeneity between OLP and non-OLP. In addition to the two loci near *CASP8* and *GFRA2* that were uniquely significant in OLP, common associations at loci containing *IFIH1*, *CEP43*, *C12orf42*, and *ZFP36L1* are more significantly associated with OLP (only the *CEP43* locus achieves even nominal significance [*p* < 0.05] in non-OLP among these six). However, the associations at the MHC and *CLEC16A* locus were more significantly associated with non-OLP. Although the MHC association is highly significant in both OLP and non-OLP, it is dramatically stronger in non-OLP (*p* = 5.6 × 10^−28^ for difference in effect), and the association in non-OLP near *CLEC16A* does not even reach nominal significance in OLP. These results indicate that the heterogeneity between the two oral and non-oral groupings does not derive from a unidirectionally larger genetic component in one subgroup than the other and that, while primarily shared, the genetic architecture of OLP and non-OLP is not identical. We colocalized eQTLs and sQTLs derived from eQTLCatalogue (https://www.ebi.ac.uk/eqtl/),^49^ pQTLs from a recent deCODE genetics study,^50^ along with published GWASs cataloged by OpenTargets^46^^,^^47^^,^^48^^,^^51^ to further inform on our genome-wide significant loci (Table 1).

### Insights from genetic associations

Unsurprisingly, the strongest association was observed to the MHC region on chromosome 6. HLA imputation was thus performed in FinnGen with HIBAG^52^ and the strongest association was observed to *DQB1^∗^05:01*. Of note, nearby HLA alleles in high linkage disequilibrium with *DQB1^∗^05:01* (specifically *DQA1^∗^01:01* and *DRB1^∗^01:01*) were more than 20 orders of magnitude less significant, suggesting *DQB1^∗^05:01* stands out as the likely relevant associated allele. Further dissection of this association using PLINK^36^ to explore the HLA-imputation data demonstrated that the risk of the *DQB1^∗^05:01* haplotype is substantially stronger in non-oral LP (OR = 2.09 [CI 2.00,2.18]) vs. oral (OR = 1.36 [CI 1.29,1.44]). *DQB1^∗^05:01*, the most common haplotype in Finland, is not associated with autoimmune risk in FinnGen,^53^ but in prior reports has been associated with 1.8-fold risk of follicular lymphoma (*p* = 6.0 × 10^−38^)^54^ and protection against pernicious anemia (*p* = 1.7 × 10^−17^).^55^ These results indicate that although there is a shared major HLA risk in *DQB1^∗^05:01*, it has a substantially different impact in the two subgroupings, further distinguishing their architectures. Of note, the *CLEC16A* locus, also seen more significantly in the non-oral subgroup, is adjacent to *CIITA*, a master regulator of MHC class 2 transcription—though no convincing link between our variant and expression or protein levels of any gene in this region has been demonstrated.

Associated variants directly implicating genes in LP pathogenesis include a low-frequency hypomorphic allele in *IFIH1* (rs35667974) well-established to be protective against many autoimmune diseases after initial report in type 1 diabetes^56^; a missense variant in *CPVL* (rs117744081) that has been previously associated with vitiligo^57^^,^^58^ and basal cell carcinoma^47^; and a protective low-frequency missense variant in *IKZF3* (rs112301322) in complete linkage disequilibrium (LD), r^2^ = 1, with previously reported associations to lower eosinophil count,^51^^,^^59^^,^^60^ as well as elevated total protein level,^61^^,^^62^^,^^63^ and risk to SLE.^64^

In addition to being related to autoimmune disease through HLA, the most significant LP hits are seen in other autoimmune diseases. Five of the most significant hits after HLA—*TNFSF11*, *CEP43*, *LPP*, *C12orf42*, and *IFIH1*—and ten overall coincide with the same direction of effect as seen in the FinnGen and/or UKBB autoimmune hypothyroidism GWAS. The strongest *LPP* association (rs6780858) is in high LD with associations reported in vitiligo^58^ and celiac disease^65^ and, in addition to autoimmune disease diagnoses, the association also colocalizes in FinnGen with hits for actinic keratosis (beta = 9.1, *p* = 2.3 × 10^−12^) and thyrotoxicosis (beta = −0.15, *p* = 3.4 × 10^−8^).

Two loci were specific to the FinnGen study and represent highly Finnish-enriched low-frequency variation that are thus not testable in UKBB. One is on 11q13.4–5 near *RPS3* and constitutes a credible set of three variants spanning 500 kb with no direct implication of any specific gene in the region. The second is a single intronic risk variant in *TNRC18* recently documented as a strong risk factor in IBD (in R11 beta = 0.79, *p* = 6.5 × 10^−142^) and other inflammatory diseases such as ankylosing spondylitis, psoriasis, and iridocyclitis,^37^ but with a significant opposite-direction protective effect in canonical autoimmune diseases such as hypothyroidism, type 1 diabetes, and multiple sclerosis.

We assessed intersection of our lead variants systematically with available GWAS results in FinnGen, UKBB, and OpenTargets (https://genetics.opentargets.org/) and with large-scale transcriptomic data from eQTL Catalog release 6 (https://www.ebi.ac.uk/eqtl/)^49^ and proteomic data from a recent deCODE genetics study.^50^ Where fine-mapping was available, we defined overlap as the presence of our lead variant in the credible set reported; when this was not available, a high correlation (r^2^ > 0.8) of our lead variant to the reported association lead variant was utilized via the OpenTargets Genetics platform curation of GWAS literature. Likely overlaps defined in this fashion are reported in Table 1 for select phenotypes and transcriptomic and proteomic resources.

Tallying non-MHC associations, we find the largest overlap (9 of our associations coincident) with reported basal eosinophil count variants. Eight of these 9 had consistent direction of effect (i.e., increasing eosinophil count corresponding to increased LP risk). Other blood cell count association data showed fewer overlaps and no similar consistency of directionality. Nine associations are directionally consistently shared between LP and autoimmune hypothyroidism, and one, the aforementioned *TNRC18* association, has an opposite direction of effect in LP compared with autoimmune hypothyroidism. Overlaps with other immune-mediated skin diseases and autoimmune diseases are also summarized in Table 1.

One noteworthy intersection with eQTL data occurs on chromosome 13q14.11, where our LP association (led by rs2093816) coincides with a *cis*-acting eQTL for *TNFSF11* in lymphoblastoid cell lines (LCLs) in both the Geuvadis consortium^66^ and the Twins UK study.^67^ The risk of lichen planus is associated with genetically mediated lower expression of *TNFSF11*, and intriguingly, several case reports suggest lichenoid reaction may be a rarely observed side effect of denosumab, a RANKL (encoded by *TNFSF11*) inhibitor that has been widely utilized for more than a decade as a treatment for osteoporosis.^68^^,^^69^ The genetic evidence here suggests a possibly similar mechanism for variants at *TNFSF11* that correlate with lower levels of *TNFSF11* and associate with a higher risk for LP. However, in our cohort, we do not see significant excess risk following denosumab treatment; 51/7,678 LP-affected individuals had denosumab in the year preceding their first LP diagnosis, compared to 2,261/367,349 sex- and birth year-matched control subjects (*p* = 0.56, OR = 1.08).

### Insights from genetic correlation and polygenic analysis

We explored the genetic correlation between the three lichen planus phenotypes (all FinnGen LP and the OLP and non-OLP subgroups) and 2,381 case-control FinnGen phenotypes. Ignoring associations between LP and closely related diagnoses, after correcting for multiple testing (significance thresholds: *p* < 0.05/(2,381^∗^3) = 6.3 × 10^−5^), we found a positive genetic correlation with chronic sinusitis (OLP: r_g_ = 0.38, *p* = 3.6 × 10^−7^), hallux valgus (all LP: r_g_ = 0.20, *p* = 5.4 × 10^−7^; OLP r_g_ = 0.27, *p* = 7.2 × 10^−6^), deviated nasal septum (all LP: r_g_ = 0.24, *p* = 3.2 × 10^−6^), soft tissue disorders (ICD10 M65-M68, OLP r_g_ = 0.32, *p* = 7.9 × 10^−6^), arthropathies (ICD10 M00-M25, OLP: r_g_ = 0.28, *p* = 8.2 × 10^−6^), and temporomandibular joint disorders (TMD) (all LP: r_g_ = 0.31, *p* = 2.4 × 10^−6^) (Table S4). No negative genetic correlations were observed. Consistent with most significant loci demonstrating association to both OLP and non-OLP, the two subgroupings showed significant genetic correlation (r_g_ = 0.825, *p* = 0.0008).

Next, we sought to investigate the association between lichen planus and polygenic scores (PGSs) for 129 traits calculated for 392,650 FinnGen individuals (R9) (Table S5). Consistent with the direct association overlaps described above, the strongest observations for the all LP group were seen positively with pan-UKBB hypothyroidism (*p* = 1.7 × 10^−21^), multiple sclerosis (*p* = 9.5 × 10^−16^),^70^ pan-UKBB psoriasis (*p* = 7.1 × 10^−12^), eosinophil counts (*p* = 1.7 × 10^−10^),^51^ multisite chronic pain (*p* = 1.1 × 10^−7^),^71^ and FinnGen IBD (*p* = 1.4 × 10^−7^) and negatively with several cognitive phenotypes. OLP was significant for hypothyroidism (*p* = 9.1 × 10^−16^), multisite chronic pain (*p* = 5.6 × 10^−6^), and pan-UKBB urate levels (*p* = 5.8 × 10^−6^), while non-OLP had no significant associations other than cognitive.

We additionally ran functional mapping and annotation of genome-wide association studies (FUMA) GENE2FUNC^72^ for the genes in genome-wide significant associations from the meta-analysis that showed evidence either via missense variant or QTL evidence (eQTL, pQTL, or sQTL) or were the nearest gene (Table 1). FUMA GTEX v.8 tissue differentially expressed gene (DEG) analysis showed a significant 2-side association with blood (adj *p* = 3.5 × 10^−3^), heart (adj *p* = 8.0 × 10^−3^), and spleen (adj *p* = 9.2 × 10^−3^) (Table S6). Consistent with our previous analyses, FUMA implicates a variety of immune-related pathways including GO_LYMPHOCYTE_ACTIVATION, GO_T_CELL_ACTIVATION, GO_REGULATION_OF_HEMOPOIESIS, GO_POSITIVE_REGULATION_OF_IMMUNE_SYSTEM_PROCESS, GO_REGULATION_OF_IMMUNE_SYSTEM_PROCESS, GO_RESPONSE_TO_CYTOKINE, and GO_LEUKOCYTE_DIFFERENTIATION, primarily through largely overlapping subsets of *IL2RA*, *ZNF365*, *RPS3*, *PLXNC1*, *TNFSF11*, *ZFP36L1*, *IQGAP1*, *IFIH1*, *CASP8*, *FOXP1*, and *RGS14*. The full list is given as Table S7.

## Discussion

This study consisted of 7,679 individuals with LP in FinnGen and an additional 1,998 from UK Biobank. The prevalence of LP was 1.6% in the Finnish data, broadly consistent with an overall reported incidence of LP of about 1% worldwide.^3^ Using diagnoses, procedure codes, and clinic of diagnosis, we divided LP-affected individuals into oral LP and non-oral subgroups (additional subgroup details and overlap descriptions in Figures 1 and 2). LP-affected individuals were more often female compared with the overall FinnGen cohort, and with an even greater excess of females among OLP versus non-oral LP, with both subgroups having the same median age of onset. These findings are in line with earlier reports on inflammatory and autoimmune traits, where higher prevalence is often seen in females.^73^ Furthermore, our rates and overlap (see material and methods) are comparable to a previous study^74^ where it was found that roughly half of LP-affected individuals presented oral lesions (53.6%), with 19% of individuals with cutaneous LP having OLP and 17% of OLP-affected individuals having cutaneous lesions. Since some individuals have LP in multiple areas, it is unsurprising that most genetic risk factors are found to be shared between subgroupings, though it is notable that the genetic findings here support that these are not identical in terms of underlying molecular pathology.

Lichen planus is one specific inflammatory disease that can affect various body areas, either concomitantly or sequentially. The histopathological appearance of LP is quite similar in different locations, but multiple facets differ in cutaneous and mucosal LP. First, the clinical forms of LP in different areas differ. The classic skin LP presents as pruritic, purple, polygonal, flat-topped (planar) papules crossed by fine white lines.^1^^,^^2^ LP lesions on non-keratinized epithelium are usually non-pruritic but often a burning sensation can be felt. Unlike in cutaneous LP, erosion can sometimes be seen in mucosal LP. In addition, the skin lesions are usually transient, but OLP is usually chronic and often more resistant to treatment than the cutaneous form.^11^^,^^74^ While OLP is more common among females, cutaneous LP has not demonstrated a significant sex bias in prior studies.^3^^,^^75^^,^^76^

Whether or not oral and non-oral forms of lichen planus are the same disease has been an ongoing question. We identified a total of 27 genome-wide significant loci, most of which are associated with both non-oral LP and OLP, but some loci are specific or have a stronger association to one subgrouping of LP (non-OLP: *HLA-DQB1*, *CLEC16A*; OLP: *IFIH1*, *CASP8*, *CEP43*, *GRAF2*, *C12orf42*, and *ZFP36L1*). One major difference between oral and non-oral subgroupings comes in the HLA region. *DQB1^∗^05:01* confers a substantially higher risk to non-oral LP than OLP (*p* < 10^−27^), though it is a significant risk factor to each.

The predominantly shared genetics point to a generally shared etiology between the OLP and non-OLP subgroupings, while the significant differences confirm the existence of, and provide pointers to, biology specific to oral and non-oral occurrences. In particular the dominant role of MHC in non-oral LP might suggest that cutaneous forms may have a more classically autoimmune nature. In particular the clear association to class II alleles led by *DQB1^∗^0501*, alongside the only other non-OLP predominant association residing near *CIITA*, the master regulator of class II gene transcription, suggests an element of immune response that, while shared, is more important to the non-oral subgroup.

In our study, OLP was associated with a significant risk for oral cancer generally (OR = 9.6, [CI 7.5–12.2], *p* = 4.5 × 10^−72^) and particularly tongue cancer (OR of 13.6 [CI 9.6–19.3], *p* = 1.8 × 10^−55^). This association has been seen in another Finnish study as well in which LP was associated with an increased risk of cancer of lip, tongue, oral cavity, esophagus, larynx, and vulva.^7^ In FinnGen, OLP has a 10-year oral cancer transformation rate of 1.9% (63/3,323), which compared to a base rate of 0.2% oral cancer rate across the cohort confirms a strong cancer risk. This rate is slightly higher than published rates but is unsurprising, as FinnGen has significant clinical ascertainment and therefore likely more individuals with severe LP and those with cancer are overrepresented. In published data, tobacco use, female sex, alcohol consumption, hepatitis virus C infection, and location on the tongue and type of the lesion (atrophic, erosive) are reported to increase the transformation risk.^9^^,^^10^ In FinnGen we find only 23/7,679 LP-affected individuals with any codes related to hepatitis C and no increase in smoking in OLP-affected individuals, indicating these are not major contributors to LP in Finland. In the FinnGen cohort no data are available on type of lesion or alcohol consumption. Individuals with OLP should be examined regularly to detect malignant lesions early and special attention should be paid to the lesions on the tongue.

Lichen planus is often referred to as an immune-mediated disease, and the identification of a major HLA contribution and direct overlap of nearly half the observed associations with the genetics of more deeply studied autoimmune diseases strongly support an immune basis for LP. Two meta-analyses reported a positive and statistically significant association between OLP and hypothyroidism^33^^,^^77^ and several studies of general LP and hypothyroidism suggest this association as well.^78^^,^^79^ A previous Finnish study noted an odds ratio for the association of hypothyroidism and OLP of 2.39,^80^ consistent with our observation (OR = 1.86, [CI 1.70–2.04], *p* = 6.58 × 10^−42^). The association between hypothyroidism and non-oral LP was significant in our data with a more modest effect (OR = 1.54, [CI 1.43–1.67], *p* = 5.09 × 10^−29^). A common genetic etiology was proposed between hypothyroidism and OLP^81^ and our study supports this, indicating variation at nine loci (*IFIH1*, *LPP*, *CEP43*, *TNRC18*, *C12orf42*, *TNFSF11*, *ST3GAL6*, *IL2RA*, and *IKZF3*) that are strongly associated with both LP and autoimmune hypothyroidism.

Unexpectedly, our study revealed a substantial, directionally consistent overlap between the genetics of eosinophil levels and lichen planus where variation increasing eosinophil levels coincides with variation increasing LP risk. A number of these overlap with autoimmune associations, but several with strong effects on eosinophil levels have little or no evidence in autoimmune disease including a missense variant at *IKZF3* and intronic variation at *ZNF365* (for which the only other significant FinnGen association is to atopic dermatitis).

The reported association with other autoimmune and skin diseases was also strongly confirmed in the FinnGen study. Our study implies that, in addition to hypothyroidism, OLP might be associated with Sjögren’s syndrome, vitiligo, and celiac disease, with non-oral LP showing a stronger association to discoid lupus, psoriasis, atopic dermatitis, and pityriasis rosea. These observations are consistent with a recent Taiwanese study of 12,427 individuals with LP where it was found that LP was significantly associated with systemic lupus erythematosus (SLE), Sjögren’s syndrome, dermatomyositis, vitiligo, and alopecia areata.^82^^,^^83^

GWASs revealed common risk variants in FinnGen data for LP shared with actinic keratosis, thyrotoxicosis, autoimmune hypothyroidism, type 1 diabetes, and psoriasis. *LPP*, a significant locus for LP, is reported to be associated with vitiligo and celiac disease.^58^^,^^65^ The missense variant in *CPVL*, seen in LP, is also associated with vitiligo.^58^ *TNRC18*, which was a variant associated with LP, is associated with IBD, ankylosing spondylitis, iridocyclitis, and psoriasis and is protective for autoimmune hypothyroidism, thyroiditis, and type 1 diabetes. We could not find previous studies that reported the association of LP and psoriasis; however, we found an association with both the phenotypes and the genetic variants in our study.

In conclusion, based on our findings, oral and non-oral lichen planus have similar genetic characteristics. However, several loci have a stronger effect or are even exclusively seen in OLP or non-OLP. LP is associated and has a shared etiology with hypothyroidism and several other autoimmune diseases. OLP has an increased risk for oral cancer, especially tongue cancer, and individuals with OLP should be examined regularly to detect malignant lesions as early as possible.

## Data and code availability

Data are available in a google cloud platform storage bucket gs://fg-publication-green-public/F_2021_001_20240508/LPR11.tar. Please install gsutil or similar software to download.

For phenotypic analysis, much of the code is only applicable to data in the FinnGen secure environment, but example R scripts can be found in https://finngen.gitbook.io/finngen-handbook/working-in-the-sandbox/which-tools-are-available/miscellaneous-helper-scripts-tools and further code is available upon request.

## Supplemental information

Document S1. Figure S1 and ethics statement

Data S1. Tables S1–S7

Data S2. FinnGen members 2023

Document S2. Article plus supplemental information
